# Mediterranean Diet Adherence and One-Year Metabolic Changes in Patients with Papillary Thyroid Cancer: An Observational Study

**DOI:** 10.3390/nu17213420

**Published:** 2025-10-30

**Authors:** Jinyoung Shin, Seok-Jae Heo, Yae-Ji Lee, Sang-Wook Kang, Yu-Jin Kwon, Ji-Won Lee

**Affiliations:** 1 Department of Family Medicine, Konkuk University Medical Center, Konkuk University School of Medicine, Seoul 05030, Republic of Korea; jyshin@kuh.ac.kr; 2 Biostatistics Collaboration Unit, Department of Biomedical Systems Informatics, Yonsei University College of Medicine, Seoul 03722, Republic of Korea; sjheo@yuh.ac; 3 Department of Biostatistics and Computing, Yonsei University, Seoul 03722, Republic of Korea; ysbiostat@yuhs.ac; 4 Department of Surgery, Yonsei University College of Medicine, Seoul 03722, Republic of Korea; oralvanco@yuhs.ac; 5 Department of Family Medicine, Yongin Severance Hospital, Yonsei University College of Medicine, Yongin 16995, Republic of Korea; 6 Department of Family Medicine, Severance Hospital, Yonsei University College of Medicine, Seoul 03722, Republic of Korea; 7 Institute for Innovation in Digital Healthcare, Yonsei University, Seoul 03722, Republic of Korea

**Keywords:** papillary thyroid cancer, Mediterranean diet, cancer survivors, insulin resistance, lipoproteins

## Abstract

**Background/Objectives:** The Mediterranean diet (MD) has been associated with favorable metabolic outcomes in the general population. However, evidence of these associations among thyroid cancer survivors remains limited. This study examined whether higher MD adherence at diagnosis is associated with longitudinal changes in insulin resistance and lipid profiles in patients with papillary thyroid cancer (PTC). **Methods:** We analyzed 345 Korean patients aged ≥20 years with histologically confirmed PTC at a tertiary hospital between April 2023 and March 2024. MD adherence at baseline (diagnosis) was assessed using the Korean Mediterranean Diet Adherence Screener and categorized into tertiles. Changes in body mass index (BMI), hemoglobin A1c (HbA1c), homeostasis model assessment of insulin resistance (HOMA-IR), metabolic score for insulin resistance (METS-IR), homeostasis model assessment of β-cell function (HOMA-β), and triglyceride-glucose (TyG) index were evaluated between baseline and one year post-diagnosis. A stratified analysis was conducted according to BMI (<25 vs. ≥25 kg/m^2^). **Results:** During the one-year follow-up, patients with PTC experienced significant reductions in BMI, HbA1c, METS-IR, HOMA-β, triglycerides, LDL-cholesterol, and the TyG index, whereas HDL-cholesterol levels increased. Patients in the high MD adherence group showed decreased HOMA-IR and increased HDL-cholesterol levels compared to those in the low adherence groups. In BMI-stratified analyses, reductions in insulin and HOMA-IR were observed only among patients with obesity in the high MD adherence group. **Conclusions:** Higher adherence to the MD at diagnosis was associated with decreases in insulin resistance markers and an increase in HDL-cholesterol levels among patients with PTC during the first year after diagnosis.

## 1. Introduction

Thyroid cancer is one of the fastest-growing malignancies worldwide, with a marked prevalence in adults younger than 50 years, likely due to westernized lifestyles and increased screening rates [1]. Papillary thyroid cancer (PTC), the most common and well-differentiated type of thyroid cancer, generally has a favorable prognosis with a 5-year survival rate of 92–96% [2,3]. Follicular and medullary thyroid cancers are also well-differentiated with good prognoses, whereas anaplastic thyroid cancer has a poor prognosis [3]. Therefore, the long life expectancy of patients with PTC implies that attention should be given to health issues beyond cancer recurrence or progression [4]. A considerable proportion of deaths among thyroid cancer survivors are due to non-cancer causes, especially cardiovascular disease, underscoring the importance of metabolic health management in survivorship care [5]. Metabolic abnormalities, such as insulin resistance, dyslipidemia, and metabolic syndrome, are increasingly recognized as critical health concerns in this population [6]. Thyroid hormones regulate pancreatic β-cell function and increase lipolysis, elevating free fatty acid levels that decrease insulin sensitivity and contribute to dyslipidemia [7]. In a Korean study with 38.5% of female patients with thyroid cancer, the prevalence of metabolic syndrome was 1.22 times higher in patients with cancer than in non-cancer controls, and insulin resistance measured using HOMA-IR was significantly elevated [8]. These findings emphasize the potential role of interventions targeting modifiable lifestyle factors, particularly diet, in reducing long-term risk.

The Mediterranean diet (MD) has been widely studied as a protective dietary pattern against metabolic syndrome owing to its emphasis on fruits, vegetables, legumes, whole grains, and unsaturated fats, e.g., olive oil, coupled with limited consumption of red meat and processed foods [9]. The MD provides low levels of saturated fatty acids and high quantities of antioxidants and dietary fiber, which collectively contribute to the beneficial effects on obesity, cardiovascular disease, and lipid metabolism in the general population [10,11]. In patients with cancer, higher adherence to the MD has been associated with reduced cancer-related fatigue [12] and improved health-related quality of life [13]. However, few studies have evaluated the associations between MD adherence after cancer diagnosis and long-term metabolic health outcomes among thyroid cancer survivors.

To address this knowledge gap, the present study investigated whether adherence to the MD is associated with longitudinal changes in metabolic indices, including insulin resistance and lipid profiles, over a one-year follow-up in patients with PTC.

## 2. Materials and Methods

### 2.1. Study Participants

We retrospectively analyzed data from 436 Korean adults diagnosed with PTC who attended the outpatient clinic at Severance Hospital between April 2023 and March 2024. The diagnosis of PTC was established according to the World Health Organization (WHO) Classification of Endocrine Tumors criteria [14] and was confirmed either by fine-needle aspiration biopsy or histopathological examination following thyroidectomy. Dietary assessments were conducted at the time of diagnosis for all patients with thyroid cancer. Individuals with non-papillary thyroid cancers (n = 91) were excluded from the study. Finally, 345 patients were included in the analysis.

The study protocol was approved by the Institutional Review Board (IRB) of Severance Hospital (IRB number: 4-2025-0319) and was conducted in accordance with the Declaration of Helsinki and relevant institutional guidelines and regulations. As this study analyzed previously collected data, the requirement for patient consent was waived because no personally identifiable information was used.

### 2.2. Dietary Assessment

Dietary assessments were routinely conducted at baseline, corresponding to the time of diagnosis. Mediterranean dietary adherence score (MDS) was assessed using the Korean Mediterranean Diet Adherence Screener (K-MEDAS), a validated 14-item questionnaire to reflect the degree of adherence to the MD in the Korean population [15]. Each item was scored as either 0 or 1, yielding a total score ranging from 0 to 14, with higher scores indicating greater adherence to the MD.

### 2.3. Clinical and Metabolic Variables

We obtained clinical and metabolic variables from patients’ electronic health records (EHR) at baseline (time of diagnosis) and again at one year post-diagnosis outpatient clinic visits. Body mass index (BMI) was calculated as weight in kilograms divided by height in meters squared (kg/m^2^). Laboratory parameters, including fasting serum glucose, hemoglobin A1c (HbA1c), insulin, total cholesterol, triglycerides, high-density lipoprotein cholesterol (HDL-C), and low-density lipoprotein cholesterol (LDL-C), were collected retrospectively. Based on these parameters, surrogate indices of insulin resistance and metabolic dysfunction were calculated using standard formulas: the homeostasis model assessment of insulin resistance (HOMA-IR) [16], the metabolic score for insulin resistance (METS-IR) [17], the homeostasis model assessment of β-cell function (HOMA-β) [18], and the triglyceride-glucose (TyG) index [19].

Metabolic syndrome was defined according to the modified criteria of the National Cholesterol Education Program Adult Treatment Panel III (NCEP ATP III), requiring the presence of at least three of the following: (1) abdominal obesity (waist circumference ≥ 90 cm in men or ≥85 cm in women, according to the Korean Society for the Study of Obesity (KSSO) criteria) [20]; (2) elevated blood pressure (systolic blood pressure; SBP ≥ 130 mmHg or diastolic blood pressure; DBP ≥ 90 mmHg); (3) elevated fasting glucose (≥100 mg/dL); (4) reduced HDL-C (<40 mg/dL in men or <50 mg/dL in women); and (5) elevated triglyceride (≥150 mg/dL) levels [21].

Lifestyle factors were collected at the time of diagnosis. Smoking status was categorized as never, former, or current. Alcohol consumption was recorded based on current drinking habits. Physical activity was evaluated using the International Physical Activity Questionnaire (IPAQ) and expressed in metabolic equivalent of task (MET) units. Medical history, including hypertension, type 2 diabetes mellitus (T2DM), dyslipidemia, and other cancers, was obtained through EHR based on the International Classification of Diseases, Tenth Revision codes and medications.

### 2.4. Statistical Analysis

Continuous variables were expressed as means ± standard deviations and categorical variables as numbers and percentages. Differences in continuous variables over a one-year observational period were calculated by subtracting the values recorded at baseline (time of diagnosis) from those obtained after one year, stratified according to the MDS tertiles (low, middle, and high). These differences were assessed using analysis of variance (ANOVA), while categorical variables were compared using chi-square tests. Linear mixed-effects models with random intercepts for participants were used to evaluate longitudinal changes in BMI, glucose, HbA1c, insulin, insulin resistance indices (HOMA-IR, METS-IR, and HOMA-β), and lipid profiles across MDS tertile groups. Time (baseline and one year) and MDS tertile group were modeled as fixed effects, with a group × time interaction term included to test whether the rate of change differed by adherence level. The estimated slopes represent the annual changes in each outcome within and between groups. All models were adjusted for age, sex, alcohol consumption, smoking status, physical activity, hypertension, T2DM, and dyslipidemia. A stratified analysis was conducted by BMI (<25 vs. ≥25 kg/m^2^) to examine the potential modifying effect of obesity status on these associations. All statistical analyses were performed using R software (version 4.4.1; R Foundation for Statistical Computing, Vienna, Austria). Linear mixed-effects models were fitted using the lme4 package, and visualizations were generated with ggplot2. Two-sided *p*-values < 0.05 were considered statistically significant.

## 3. Results

### 3.1. Baseline Characteristics of Study Participants

A total of 345 patients with PTC were included in the analysis (Table 1). The mean age was 44.4 ± 11.9 years, and 71.3% of the participants were women. At baseline, the mean BMI was 24.5 ± 4.1 kg/m^2^, and 39.8% of the patients were classified as obese. Supplementary Figure S1 shows the distribution of K-MEDAS responses among the study participants. Patients in the high MDS group were significantly older and reported lower alcohol consumption than those in the low and middle MDS groups (*p* < 0.001). The prevalence of T2DM and metabolic syndrome was also higher in the high MDS group than in the low MDS group (16.8% vs. 3.7% and 40.9% vs. 27.2%, respectively). No significant differences in sex distribution, blood pressure, BMI, or dyslipidemia were observed across the three groups. The high MDS group had a higher proportion of patients who underwent total thyroidectomy and received levothyroxine replacement therapy.

### 3.2. Metabolic Changes at One Year Post Diagnosis

Table 2 corroborates these findings, showing that the mean HDL-C increased from 56.1 ± 14.9 mg/dL at baseline to 59.7 ± 15.0 mg/dL at one year, while triglycerides decreased from 129.8 ± 81.9 mg/dL to 113.7 ± 73.4 mg/dL. Collectively, these results demonstrate that higher adherence to the MD was consistently associated with improved insulin resistance and lipid profiles in patients with PTC. Thyroid hormone levels did not differ significantly across MDS tertile groups at diagnosis or at the one-year follow-up.

As shown in Table 3, several metabolic parameters significantly improved over the one-year follow-up. In the overall cohort, BMI decreased by −0.28 kg/m^2^ (95% CI, −0.53 to −0.03), HbA1c by −0.12% (95% CI, −0.21 to −0.04), METS-IR by −1.40 (95% CI, −1.90 to −0.90), HOMA-β by −10.42 (95% CI, −20.76 to −0.08), triglycerides by −17.15 mg/dL (95% CI, −27.49 to −6.81), LDL-C by −4.98 mg/dL (95% CI, −9.57 to −0.39), and the TyG index by −0.12 (95% CI, −0.19 to −0.05), whereas HDL-C increased by +4.10 mg/dL (95% CI, 2.80 to 5.41). When stratified according to MDS tertiles, patients in the high MDS group exhibited more significant changes during the observation period, including greater reductions in METS-IR, triglycerides, and TyG index, as well as a more pronounced increase in HDL-C (*p* < 0.005). Although overall changes in HOMA-IR were not statistically significant (−0.21, 95% CI, −0.58 to 0.17), compared with low (*p* = 0.036) and mid MDS groups (*p* = 0.047), the high MDS group showed a greater decrease in HOMA-IR.

### 3.3. Subgroup Analysis for BMI

**Figure 1:**
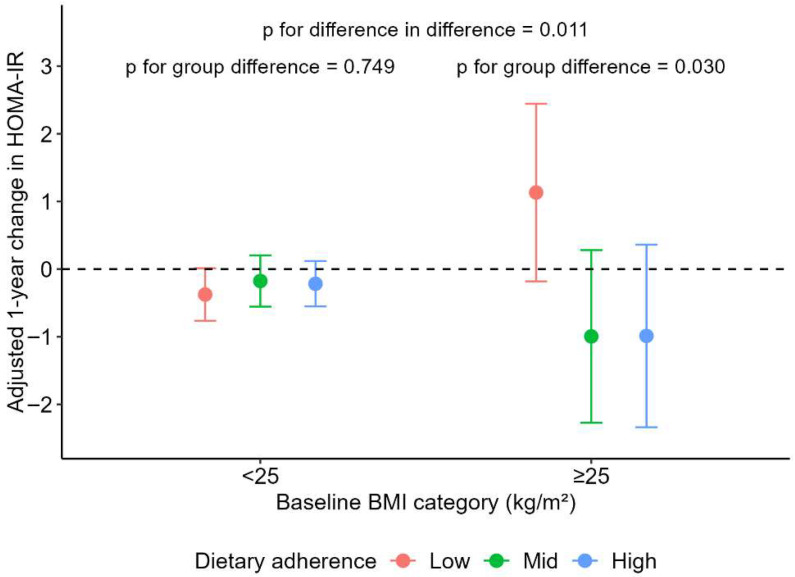
Adjusted one-year changes in HOMA-IR according to MDS tertile groups and baseline BMI category. Values represent model-estimated means ± 95% confidence intervals derived from linear mixed-effects models adjusted for age, sex, alcohol consumption, smoking status, physical activity, hypertension, T2DM, and dyslipidemia. *p*-values indicate between-group differences and group × BMI interaction effects.

In the BMI-stratified analysis (Table 4), significant metabolic changes across MDS tertiles were observed primarily among participants with obesity (BMI ≥ 25 kg/m^2^). Specifically, in this group, METS-IR showed significant improvement across all MDS levels, triglyceride decreased in high MDS groups, HDL-C significantly increased in the middle and high MDS groups, and the TyG index significantly decreased in the high MDS group. When comparing changes across MDS tertiles, insulin and HDL-C exhibited significant differences, and the reduction in insulin levels in participants with obesity remained statistically significant compared with those in the BMI < 25 kg/m^2^ group. High MDS was also associated with a decrease in HOMA-IR among participants with BMI ≥ 25 kg/m^2^, whereas this trend was not observed in those with BMI < 25 kg/m^2^ (*p* = 0.011) (Figure 1). In contrast, among participants with BMI < 25 kg/m^2^, only HDL-C showed a significant increase in the high MDS group, and no significant differences across MDS tertiles were observed (all *p*-values > 0.05).

## 4. Discussion

In this study, we found that higher adherence to the MD at diagnosis was associated with decreases in METS-IR, triglycerides, and TyG index, and an increase in HDL-C levels among patients with PTC during the first year after diagnosis. This period is typically characterized by intensive treatment regimens and patients’ efforts to adopt healthier dietary habits [22]. Consistent with this, our participants showed overall improvements in glucose, lipid, and insulin resistance markers during follow-up. Importantly, these associations were most pronounced in participants with obesity and high MD adherence, who demonstrated significant improvements in insulin resistance indices and lipid profiles. Given that the mean baseline MDS was relatively low (4.6 ± 2.0), our findings highlight the potential clinical relevance of increasing MD adherence to achieve meaningful metabolic changes, particularly with respect to insulin resistance and HDL-C levels.

Although dietary modifications after diagnosis have been reported among patients with gastrointestinal cancers [23], evidence remains limited for thyroid cancer survivors, with most available data restricted to questionnaire-based surveys in breast or ovarian cancer populations [22,24]. An 80-patient cross-sectional study in colorectal cancer confirmed that participants with high adherence to the MD exhibited significantly higher HDL-C levels, suggesting that increased MD adherence may improve lipid profiles in non-thyroid cancer populations as well [25]. By directly measuring longitudinal changes in metabolic indices rather than relying solely on self-reported perceptions, our study provides novel evidence that adherence to the MD can positively influence metabolic outcomes in thyroid cancer survivors. The MD’s anti-inflammatory and antioxidant properties—mediated by a high intake of polyphenols, omega-3 fatty acids, and dietary fiber—may reduce systemic inflammation and oxidative stress, thereby improving insulin sensitivity [11]. Moreover, the MD may modulate adipocytokines such as adiponectin and leptin, which influence insulin sensitivity and lipid metabolism [11], and could help enhance thyroid function in both euthyroid and hypothyroid status after thyroidectomy [26]. Collectively, these findings highlight the potential role of dietary counseling as a practical component of survivorship care aimed at improving long-term metabolic outcomes in thyroid cancer survivors.

Patients with cancer are often aware of the importance of healthy dietary patterns and may attempt to improve their eating habits after diagnosis to support treatment, recovery, and recurrence prevention [27]. For example, the ECHO study reported that 3% of 684 patients with breast cancer transitioned to an MD after diagnosis [28], and 33–36% of patients with colorectal cancer reported dietary changes within 12 months [29]. Although up to 84% of survivors express an intention to adopt healthier diets, sustained adherence remains challenging. Common obstacles include fatigue from meal preparation, psychological stress, and treatment-related factors such as altered taste, appetite loss, or cravings for unhealthy foods [30]. A large U.S. study of 1971 cancer survivors using the Healthy Eating Index reported generally poor diet quality, with less than 50% adherence to components overlapping with the MD, such as whole grains, greens, and beans [31]. Higher MD adherence has been associated with improvements in body weight regulation, insulin sensitivity, oxidative stress markers, and lipid profiles, contributing to reduced long-term cardiovascular and thyroid cancer risks [11,32,33]. Therefore, targeted and sustainable dietary strategies that integrate structured nutritional education into early post-diagnosis care could be effective in optimizing survivorship outcomes for thyroid cancer patients.

In our study, higher MD adherence was associated with reduced insulin resistance and increased HDL-C levels, particularly among participants with obesity. This finding is consistent with previous reports linking obesity and adverse metabolic profiles to aggressive thyroid cancer features. For instance, a higher BMI has been associated with an increased risk of lymph node metastases (OR 1.077, 95% CI: 1.013–1.145), and an elevated TG/HDL-C ratio has been linked to aggressive histological variants of differentiated thyroid carcinoma (OR 1.269, 95% CI: 1.001–1.61) [34]. As obesity and insulin resistance are well-recognized risk factors for thyroid cancer [35,36], the significant metabolic changes with higher MD adherence may be particularly pronounced in individuals with obesity compared with those without. These greater changes may be partly explained by enhanced adipokine regulation and improved lipid metabolism, which collectively contribute to better insulin sensitivity and HDL-C elevation in individuals with obesity.

However, several points should be considered when interpreting our findings. First, although HDL-C levels significantly increased across MDS groups, the clinical implications of these changes should be interpreted with caution, as even participants in the low MDS group had baseline HDL-C levels within the normal range. Second, compared with the patients in the lower adherence groups, those in the high MD adherence group were older and had a higher prevalence of T2DM and metabolic syndrome, which resulted in higher mean glucose levels. They may have used higher rates of statins and metformin, which could have influenced lipid profiles and insulin resistance. Although we adjusted for comorbidities and medication use confirmed through EHR, potential residual confounding cannot be completely ruled out. Moreover, older individuals tended to have higher MD adherence and may possess greater health awareness and motivation to adopt healthy behaviors [37,38], which could have contributed to the observed metabolic improvements. Third, dietary habits and food availability in Korea differ from those in Mediterranean regions, which may influence both the level and pattern of adherence. The traditional Korean diet shares features with the MD—such as high consumption of grains, vegetables, legumes, and fish—but also differs in its high white rice intake and relatively low fat consumption [15]. Understanding these cultural and regional dietary differences is essential for interpreting our findings. Finally, although the MDS was assessed only at diagnosis, it likely reflects habitual pre-diagnostic dietary patterns, as the K-MEDAS captures usual food intake over time. However, the lack of longitudinal data limits the evaluation of long-term adherence effects.

This study has some limitations. First, participants were recruited from one of the largest hospitals in Seoul, Korea. However, as it was conducted at a single institution, the findings may not be generalizable to all thyroid cancer survivors across diverse healthcare settings. Second, measuring MD adherence only at baseline may not accurately reflect dietary intake during the follow-up period when metabolic changes occur. Although patients’ perceptions and preferences regarding diet were likely reflected at baseline, this single-time assessment may have been insufficient to explain the metabolic changes observed after one year. Future studies with larger and more diverse cohorts are warranted to evaluate longitudinal changes using repeated dietary assessments and to determine whether sustained or changing adherence to the MD over time influences metabolic outcomes in thyroid cancer survivors. Third, as dietary intake was measured using self-reported questionnaires, recall bias or socially desirable responses may have occurred, potentially leading to overestimation of adherence. In addition, baseline health status, unmeasured lifestyle factors (e.g., physical activity, overall diet quality), and differences in the management of comorbidities such as T2DM and dyslipidemia were not fully captured, which may have contributed to residual confounding. Future studies incorporating objective lifestyle measurements and detailed treatment information are warranted to better clarify the independent associations between MD adherence and metabolic outcomes.

Despite these limitations, this study has notable strengths. It examined dynamic changes in metabolic indicators during the first year after cancer diagnosis, a critical period characterized by intensive treatments and lifestyle adjustments, based on a large sample size and the inclusion of comprehensive biochemical measurements. These strengths provide valuable insights into the potential role of dietary adherence in metabolic adaptation and survivorship care during this transitional phase.

## 5. Conclusions

This study demonstrated that higher adherence to the MD is associated with decreases in insulin resistance and an increase in HDL-C levels among patients with PTC, particularly those with obesity. These findings highlight the potential role of adopting healthy dietary patterns to mitigate adverse metabolic profiles and enhance long-term thyroid cancer survivorship care. Future prospective multicenter studies are warranted to confirm these associations and establish effective, evidence-based dietary strategies for improving metabolic health in thyroid cancer survivors.

## Figures and Tables

**Table 1:** Baseline characteristics of study participants according to MDS tertile at diagnosis of PTC.

Variable	Overall (n = 345)	Low (n = 107)	Mid (n = 119)	High (n = 119)	*p*-Value
Age (years)	44.4 ± 11.9	39.4 ± 9.6	45.0 ± 12.2	48.3 ± 11.9	<0.001
Sex					0.943
Male	99 (28.7%)	32 (29.9%)	34 (28.6%)	33 (27.7%)	
Female	246 (71.3%)	75 (70.1%)	85 (71.4%)	86 (72.3%)	
SBP (mmHg)	120.6 ± 15.5	118.7 ± 19.4	121.5 ± 12.9	121.3 ± 13.8	0.415
DBP (mmHg)	77.2 ± 9.9	76.7 ± 10.2	77.1 ± 9.4	77.8 ± 10.1	0.751
BMI classification					0.411
Underweight	11 (3.2%)	3 (2.8%)	1 (0.8%)	7 (5.9%)	
Normal	132 (38.4%)	39 (36.4%)	50 (42.0%)	43 (36.4%)	
Overweight	64 (18.6%)	22 (20.6%)	19 (16.0%)	23 (19.5%)	
Obese	137 (39.8%)	43 (40.2%)	49 (41.2%)	45 (38.1%)	
Alcohol consumption, yes (%)	164 (47.5%)	63 (58.9%)	61 (51.3%)	40 (33.6%)	<0.001
Smoking status, yes (%)	16 (4.6%)	9 (8.4%)	4 (3.4%)	3 (2.5%)	0.100
Physical activity, yes (%)	143 (42.6%)	35 (34.7%)	50 (43.1%)	58 (48.7%)	0.110
HTN, yes (%)	107 (31.0%)	28 (26.2%)	34 (28.6%)	45 (37.8%)	0.134
T2DM, yes (%)	35 (10.1%)	4 (3.7%)	11 (9.2%)	20 (16.8%)	0.004
Dyslipidemia, yes (%)	141 (40.9%)	38 (35.5%)	45 (37.8%)	58 (48.7%)	0.095
Metabolic syndrome, yes (%)	102 (30.9%)	28 (27.2%)	27 (24.1%)	47 (40.9%)	0.016
Abdominal obesity	214 (62.2%)	68 (63.6%)	74 (62.2%)	72 (61.0%)	0.922
Increased blood pressure	113 (32.8%)	31 (29.2%)	38 (31.9%)	44 (37.0%)	0.473
Increased fasting glucose	133 (38.9%)	33 (30.8%)	40 (34.5%)	60 (50.4%)	0.006
Decreased HDL-C	84 (25.1%)	22 (21.0%)	24 (20.9%)	38 (33.0%)	0.061
Increased triglycerides	91 (28.4%)	30 (30.6%)	24 (21.8%)	37 (33.0%)	0.147
Thyroidectomy					0.063
Partial	234 (67.8%)	77 (72.0%)	84 (70.6%)	73 (61.3%)	
Total	111 (32.2%)	30 (28.0%)	35 (29.4%)	46 (38.7%)	
Radioactive iodine therapy	81 (23.5%)	21 (19.6%)	26 (21.8%)	34 (28.6%)	0.251
Levothyroxine replacement	336 (97.4%)	102 (95.3%)	115 (96.6%)	119 (100.0%)	0.047

Data are presented as numbers (%) or mean ± standard deviation. PTC: papillary thyroid cancer, SBP: systolic blood pressure, DBP: diastolic blood pressure, BMI: body mass index, HTN: hypertension, T2DM: type 2 diabetes mellitus, and HDL-C: high-density lipoprotein cholesterol.

**Table 2:** Clinical and metabolic variables in patients with PTC according to MDS at diagnosis and one year post-diagnosis.

Variable	At Diagnosis					Post-Diagnosis for One Year				
	Overall (n *=* 345)	Low (n *=* 107)	Mid (n *=* 119)	High (n *=* 119)	*p*-Value	Overall (n *=* 345)	Low (n *=* 107)	Mid (n *=* 119)	High (n *=* 119)	*p*-Value
BMI	24.5 ± 4.1	24.5 ± 4.3	24.4 ± 3.8	24.5 ± 4.3	0.987	24.3 ± 4.0	24.5 ± 4.1	24.5 ± 3.9	23.9 ± 3.9	0.671
Glucose	99.9 ± 12.8	97.4 ± 9.0	99.3 ± 13.4	102.8 ± 14.5	0.005	100.1 ± 13.2	99.1 ± 14.7	99.5 ± 11.7	101.8 ± 13.1	0.29
HbA1c	6.1 ± 0.7	5.7 ± 0.3	6.0 ± 0.7	6.4 ± 0.8	0.021	5.6 ± 0.4	5.6 ± 0.3	5.6 ± 0.4	5.7 ± 0.5	0.178
Insulin	10.8 ± 7.9	10.3 ± 5.5	10.9 ± 9.3	11.2 ± 8.2	0.705	9.4 ± 9.0	11.3 ± 13.6	8.2 ± 6.2	8.9 ± 4.9	0.219
HOMA-IR	2.7 ± 2.2	2.5 ± 1.4	2.7 ± 2.7	2.9 ± 2.2	0.412	2.4 ± 2.5	2.9 ± 3.9	2.1 ± 1.6	2.2 ± 1.3	0.288
METS-IR	36.1 ± 9.7	35.8 ± 8.5	36.2 ± 11.8	36.3 ± 8.5	0.928	34.6 ± 7.6	34.3 ± 8.3	35.0 ± 7.8	34.4 ± 6.7	0.885
HOMA-β	110.3 ± 86.5	110.5 ± 60.1	112.8 ± 102.1	107.6 ± 90.1	0.903	94.3 ± 70.4	112.9 ± 95.3	80.4 ± 55.6	91.0 ± 51.5	0.072
Total Cholesterol	190.7 ± 38.2	192.0 ± 36.5	191.6 ± 39.3	188.7 ± 38.9	0.784	190.3 ± 37.4	191.8 ± 31.8	188.5 ± 38.6	190.8 ± 40.8	0.818
Triglycerides	129.8 ± 81.9	131.1 ± 93.8	122.4 ± 78.1	136.0 ± 74.1	0.457	113.7 ± 73.4	115.6 ± 66.2	108.6 ± 65.1	117.9 ± 88.5	0.743
HDL-C	56.1 ± 14.9	58.2 ± 14.8	56.3 ± 14.9	53.9 ± 14.7	0.106	59.7 ± 15.0	60.5 ± 14.2	59.1 ± 16.3	59.7 ± 14.3	0.871
LDL-C	116.3 ± 33.5	120.2 ± 34.0	116.6 ± 33.8	112.6 ± 32.7	0.264	112.5 ± 33.1	116.6 ± 34.0	111.5 ± 33.1	109.6 ± 32.5	0.487
TyG	8.6 ± 0.6	8.6 ± 0.6	8.6 ± 0.6	8.7 ± 0.6	0.071	8.5 ± 0.5	8.5 ± 0.6	8.5 ± 0.5	8.5 ± 0.6	0.855
T3	0.94 ± 0.19	0.94 ± 0.15	0.95 ± 0.19	0.94 ± 0.21	0.883	1.00 ± 0.21	1.03 ± 0.20	0.98 ± 0.22	1.00 ± 0.19	0.276
Free T4	1.01 ± 0.23	0.99 ± 0.10	1.00 ± 0.11	1.04 ± 0.37	0.418	1.09 ± 0.18	1.08 ± 0.17	1.08 ± 0.18	1.10 ± 0.19	0.654
TSH	1.87 ± 1.47	1.92 ± 1.56	1.74 ± 1.03	1.94 ± 1.74	0.419	1.85 ± 6.68	1.14 ± 1.26	1.69 ± 4.40	2.63 ± 10.31	0.149

Values are mean ± SD. *p*-values were obtained from analysis of variance. PTC: papillary thyroid cancer, MDS: Mediterranean dietary adherence score, BMI: body mass index, HbA1c: hemoglobin A1c, HOMA-IR: homeostasis model assessment of insulin resistance, METS-IR: metabolic score for insulin resistance, HOMA-β: homeostasis model assessment of β-cell function, HDL-C: high-density lipoprotein cholesterol, LDL-C: low-density lipoprotein cholesterol, TyG: triglyceride-glucose index.

**Table 3:** Metabolic changes from diagnosis to one year post-diagnosis based on the MDS tertiles.

Variables	Coefficient (95% CI) for Change				*p*-Value		
	Overall (n = 345)	Low MDS (n = 107)	Mid MDS (n = 119)	High MDS (n = 119)	Low vs. Mid	Low vs. High	Mid vs. High
BMI	−0.28 (−0.53, −0.03) *	−0.34 (−0.78, 0.11)	−0.36 (−0.77, 0.06)	−0.14 (−0.57, 0.29)	0.954	0.531	0.998
Glucose	0.02 (−1.21, 1.25)	1.58 (−0.69, 3.86)	−0.12 (−2.20, 1.96)	−1.11 (−3.16, 0.95)	0.279	0.085	0.303
HbA1c	−0.12 (−0.21, −0.04) *	−0.05 (−0.23, 0.13)	−0.17 (−0.31, −0.03) *	−0.121 (−0.26, 0.02)	0.313	0.551	0.272
Insulin	−0.92 (−2.23, 0.39)	1.13 (−1.21, 3.48)	−1.65 (−3.93, 0.63)	−2.03 (−4.22, 0.17)	0.095	0.054	0.062
HOMA-IR	−0.21 (−0.58, 0.17)	0.44 (−0.22, 1.11)	−0.45 (−1.10, 0.19)	−0.53 (−1.15, 0.09)	0.058	0.036	0.047
METS-IR	−1.40 (−1.90, −0.90) *	−1.27 (−2.17, −0.36) *	−1.23 (−2.07, −0.39) *	−1.69 (−2.57, −0.82) *	0.953	0.508	0.691
HOMA-β	−10.42 (−20.76, −0.08) *	0.27 (−18.25, 18.80)	−14.95 (−33.08, 3.18)	−15.61 (−32.94, 1.72)	0.250	0.220	0.140
Total Cholesterol	−0.82 (−5.11, 3.47)	−2.10 (−10.06, 5.85)	−3.13 (−10.40, 4.14)	2.47 (−4.71, 9.64)	0.852	0.403	0.869
Triglycerides	−17.15 (−27.49, −6.81) *	−15.95 (−34.80, 2.89)	−15.68 (−32.85, 1.48)	−19.62 (−37.71, −1.53) *	0.983	0.783	0.884
HDL-C	4.10 (2.80, 5.41) *	2.51 (0.18, 4.84) *	3.29 (1.16, 5.41) *	6.54 (4.28, 8.80) *	0.629	0.015	0.040
LDL-C	−4.98 (−9.57, −0.39) *	−5.26 (−13.60, 3.07)	−5.05 (−12.66, 2.55)	−4.71 (−12.78, 3.35)	0.971	0.926	0.751
TyG index	−0.12 (−0.19, −0.05) *	−0.08 (−0.20, 0.05)	−0.09 (−0.20, 0.03)	−0.19 (−0.31, −0.07) *	0.898	0.190	0.726

* *p*-value < 0.005, Values are regression coefficients with 95% confidence intervals estimated from linear mixed-effects models. Each coefficient represents the adjusted mean 1-year change in the corresponding variable. Models were adjusted for age, sex, alcohol consumption, smoking status, physical activity, hypertension, T2DM, and dyslipidemia. MDS: Mediterranean dietary adherence score, BMI: body mass index, HbA1c: hemoglobin A1c, HOMA-IR: homeostasis model assessment of insulin resistance, METS-IR: metabolic score for insulin resistance, HOMA-β: homeostasis model assessment of β-cell function, HDL-C: high-density lipoprotein cholesterol, LDL-C: low-density lipoprotein cholesterol, TyG: triglyceride-glucose.

**Table 4:** BMI-stratified analysis of metabolic changes over one year following diagnosis by MDS tertile groups.

	BMI < 25 kg/m^2^			*p*-Value	BMI ≥ 25 kg/m^2^			*p*-Value	*p* for DID
	Low	Mid	High		Low	Mid	High		
Glucose	−0.42 (−2.90, 2.07)	0.75 (−1.46, 2.97)	−0.66 (−2.81, 1.49)	0.638	3.95 (−0.09, 7.99)	−1.37 (−5.18, 2.44)	−0.99 (−4.89, 2.90)	0.115	0.122
Insulin	−1.53 (−3.04, −0.01) *	−0.74 (−2.22, 0.74)	−0.93 (−2.24, 0.37)	0.753	3.30 (−1.18, 7.78)	−3.24 (−7.59, 1.12)	−3.87 (−8.48, 0.74)	0.043	0.021
METS-IR	−0.68 (−1.68, 0.33)	−0.26 (−1.28, 0.76)	−0.88 (−1.82, 0.05)	0.659	−2.08 (−3.54, −0.63) *	−2.37 (−3.60, −1.14) *	−3.12 (−4.60, −1.63) *	0.575	0.316
HOMA-β	−12.83 (−29.30, 3.64)	−7.37 (−23.44, 8.70)	−11.37 (−25.52, 2.79)	0.893	10.52 (−24.02, 45.06)	−26.70 (−60.56, 7.17)	−25.95 (−61.73, 9.83)	0.214	0.327
Total cholesterol	1.64 (−8.67, 11.94)	−1.79 (−10.95, 7.38)	8.87 (−0.03, 17.77)	0.249	−6.46 (−18.86, 5.95)	−4.57 (−16.26, 7.13)	−7.12 (−19.09, 4.85)	0.953	0.508
Triglycerides	−21.78 (−44.12, 0.57)	−16.66 (−37.92, 4.61)	−12.514 (−32.88, 7.85)	0.830	−7.26 (−38.92, 24.40)	−18.67 (−46.25, 8.91)	−24.59 (−57.88, 8.69)	0.731	0.364
HDL-C	3.16 (−0.32, 6.64)	2.60 (−0.82, 6.03)	5.91 (2.67, 9.14) *	0.331	1.72 (−1.30, 4.73)	4.32 (1.77, 6.87) *	7.35 (4.18, 10.51) *	0.039	0.860
LDL-C	−4.40 (−15.93, 7.14)	−1.63 (−12.66, 9.40)	−0.53 (−11.09, 10.03)	0.887	−5.18 (−17.37, 7.01)	−9.38 (−19.99, 1.24)	−12.54 (−25.48, 0.40)	0.698	0.812
TyG	−0.15 (−0.30,−0.003) *	−0.11 (−0.25, 0.03)	−0.13 (−0.26, 0.01)	0.911	0.03 (−0.19, 0.23)	−0.10 (−0.28, 0.08)	−0.26 (−0.48, −0.04) *	0.165	0.086

Values are regression coefficients with 95% confidence intervals estimated from linear mixed-effects models stratified by baseline BMI category (<25 or ≥25 kg/m^2^). Each coefficient represents the adjusted mean one-year change in the corresponding variable within each MDS tertile group. Asterisks (*) indicate statistically significant differences (*p* < 0.05). *p*-value indicates the between-group difference within each BMI category, and *p* for DID tests whether the group-related change differs by BMI category (interaction effect). Models were adjusted for age, sex, alcohol consumption, smoking status, physical activity, hypertension, T2DM, and dyslipidemia. BMI: body mass index, DID: difference-in-difference, METS-IR: metabolic score for insulin resistance, HOMA-β: homeostasis model assessment of β-cell function, HDL-C: high-density lipoprotein cholesterol, LDL-C: low-density lipoprotein cholesterol, TyG: triglyceride-glucose index.

## Data Availability

The data analyzed in the current study are available from the corresponding authors upon reasonable request. The data are not publicly available due to being a part of an ongoing study and are part of an institutional database accessible only to the research team of the hospital.

## Supplementary Materials

The following supporting information can be downloaded at: https://www.mdpi.com/article/10.3390/nu17213420/s1, Figure S1: The response of K-MEDAS questions among the study participants (n = 345).

## Abbreviations

The following abbreviations are used in this manuscript:

BMI	Body mass index
DBP	Diastolic blood pressure
K-MEDAS	Korean Mediterranean Diet Adherence Screener
LDL-C	Low-density lipoprotein cholesterol
HbA1c	Hemoglobin A1c
HDL-C	High-density lipoprotein cholesterol
HOMA-IR	Homeostasis model assessment of insulin resistance
HOMA-β	Homeostasis model assessment of β-cell function
MD	Mediterranean diet
MDS	Mediterranean dietary adherence score
METS-IR	Metabolic score for insulin resistance
PTC	Papillary thyroid cancer
SBP	Systolic blood pressure
TyG	Triglyceride-glucose
