# Supplementary figures and images for: Mediterranean Diet Adherence and One-Year Metabolic Changes in Patients with Papillary Thyroid Cancer: An Observational Study
Source: Nutrients. 2025 Oct 30;17(21):3420. doi: 10.3390/nu17213420 (PMC12611023; doi:10.3390/nu17213420)

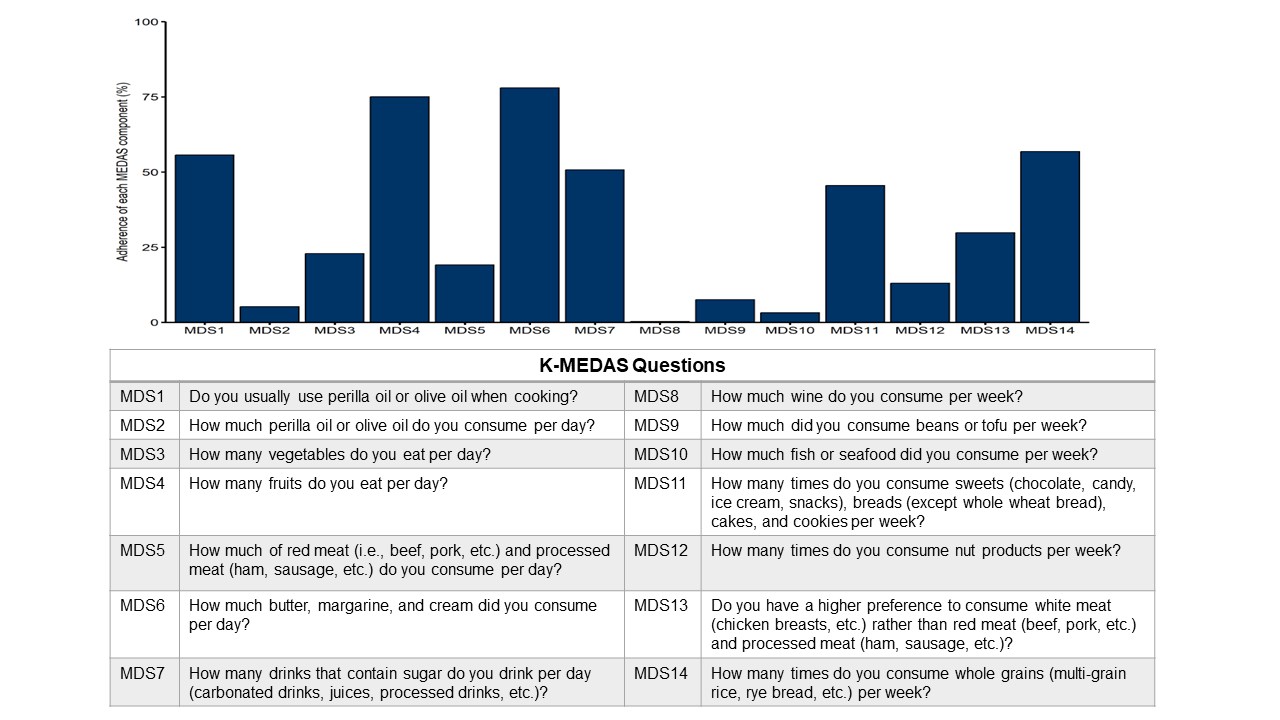

Supplement: Supplementary file 1 [file nutrients-17-03420-s001.zip › nutrients-3920822-supplementary.png]
